# Role of Cardiac Magnetic Resonance (CMR) as a Diagnostic and Risk Stratification Tool for a Patient With Arrhythmic Mitral Valve Complex Phenotype and Tricuspid Annular Disjunction (TAD)

**DOI:** 10.7759/cureus.104435

**Published:** 2026-02-28

**Authors:** Mahmoud Raslan, Xi Na Huang, Zeyad M Elmarzouky

**Affiliations:** 1 Cardiology, Southampton General Hospital, Southampton, GBR; 2 Cardiology, Gloucestershire Royal Hospital, Gloucester, GBR

**Keywords:** arrhythmogenic mitral valve complex, cardiac mri, mitral annular disjunction, mitral valve prolapse, tricuspid annular disjunction

## Abstract

Arrhythmogenic mitral valve disease (AMVD) is a newly established entity that carries a high risk of developing ventricular arrhythmia ranging from premature ventricular contraction to sudden cardiac death. Therefore, there is an urgent need for risk prediction and stratification by using different imaging modalities.

A 77-year-old patient presented with a syncopal episode. Physical examination was unremarkable. Resting electrocardiogram (ECG) was normal. Echocardiography showed mild systolic dysfunction and mild aortic stenosis. Seven days of Holter monitor recording demonstrated frequent episodes of polymorphic ventricular contraction and one episode of sinus bradycardia. Therefore, the patient was scheduled for cardiac magnetic resonance (CMR) which was successful in revealing the etiology of the presentation. Biannuli disjunction and mitral valve prolapse could be clearly visualized in our CMR study. Consequently, the patient was referred for an urgent clinical consultation by the electrophysiology heart team and implantable loop recorder. A review of the six-week recording showed further episodes of narrow complex tachycardia with rates up to 222 beats per minute (bpm) associated with one episode of pre-syncope. He was prescribed bisoprolol and scheduled for regular follow-up at the arrhythmia clinic.

In our report, we present a case of AMVD when the definite diagnosis could not be revealed by using the echocardiography modality alone. However, CMR has been proven successful in establishing a diagnosis and unveiling the etiology of the presentation.

## Introduction

Mitral annular disjunction (MAD) is defined as the abnormal systolic displacement of the atrial wall-mitral junction and left ventricle (LV) free wall [1]. There is a common association between MAD and mitral valve prolapse (MVP) [2,3]. Both conditions, together or independently, have been reported to be highly associated with ventricular arrhythmia (VA) and sudden cardiac death (SCD) [4,5]. Similarly, tricuspid annular disjunction (TAD) demonstrates the abnormal separation between the tricuspid valve (TV) leaflet and right ventricle (RV) basal myocardium during systole [6]. Although a recent study found that TAD is a commonly associated finding in patients presenting with MAD [7], there is no significant correlation between TAD and VA [8]. MAD and TAD could be identified on transthoracic echocardiography (TTE) and transesophageal echocardiography (TEE) [9]. However, cardiac magnetic resonance (CMR) has a higher sensitivity to detect small MAD <4 mm [10,11] and TAD as well [6]. In our report, we describe a case of bilateral MAD and TAD in a patient presenting with syncope. Furthermore, we are trying to highlight the role of CMR as a diagnostic and guidance tool for arrhythmic risk stratification and also outlining the clinical decision in symptomatic patients with arrhythmogenic mitral valve disease (AMVD) phenotype.

## Case presentation

A 77-year-old male patient presented to our syncope clinic with one episode of syncope and a few pre-syncopal attacks. Physical examination was normal, and resting electrocardiogram (ECG) revealed a normal sinus rhythm of 92 beats per minute (bpm) without ectopic beats. Blood pressure was normal at 130/70 mmHg with no orthostatic hypotension. There were no signs of fluid overload, and cardiac auscultation showed normal but distant heart sounds without any murmur. The patient had a TTE, which was difficult to interpret due to the high burden of ventricular ectopy and poor echocardiographic windows. Nevertheless, it showed mildly impaired LV systolic function and trivial mitral valve regurgitation during systole, as seen in Video 1.

**Video 1:** Two-dimensional echocardiography, apical three-chamber view with color Doppler, showing trivial mitral valve regurgitation during systole

**Figure 1:**
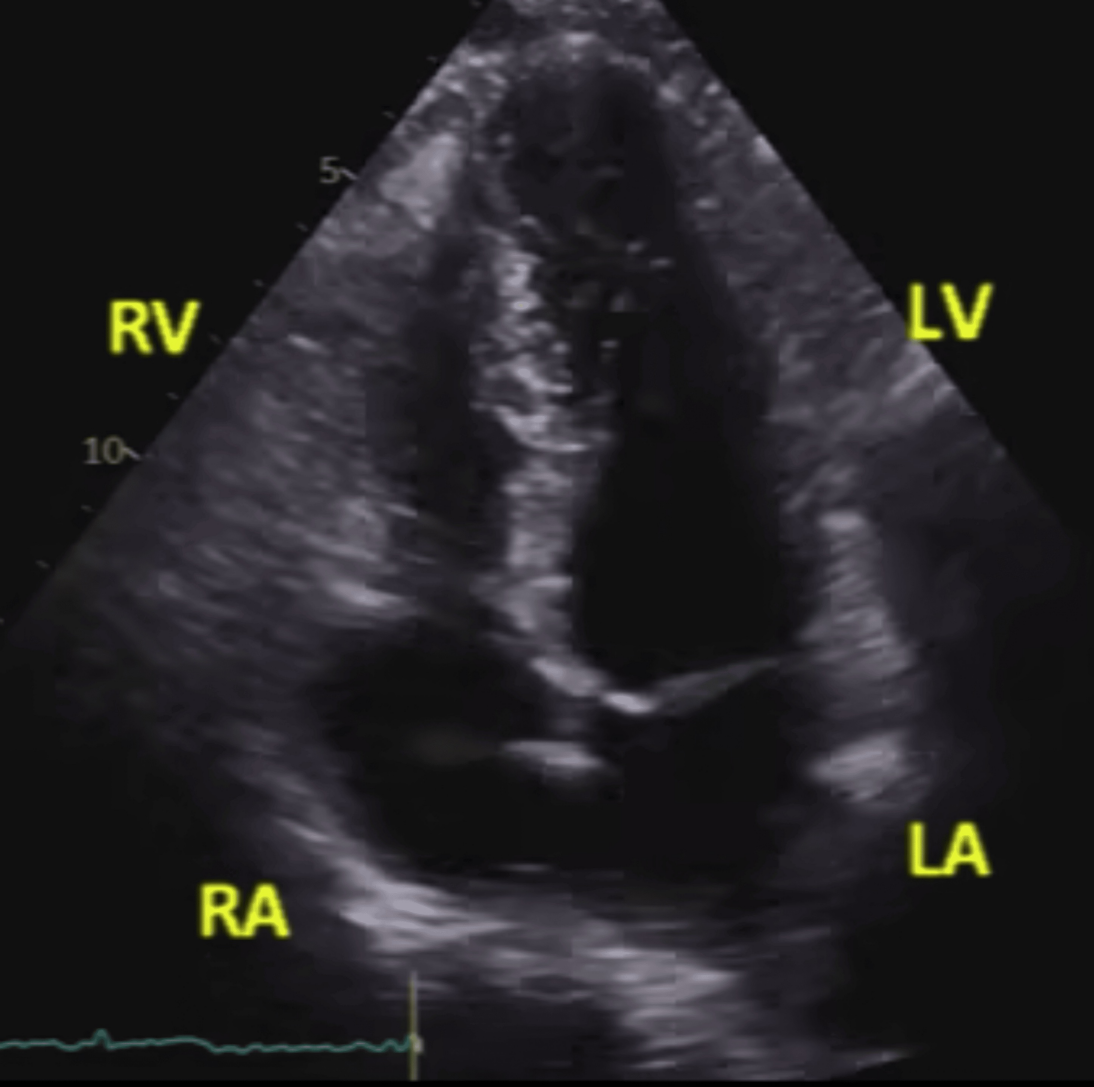
Two-dimensional transthoracic echocardiography, apical four-chamber view, showing a static frame of the mitral and tricuspid annuli during systole without obvious displacement RV: right ventricle; LV: left ventricle; RA: right atrium; LA: left atrium

**Figure 2:**
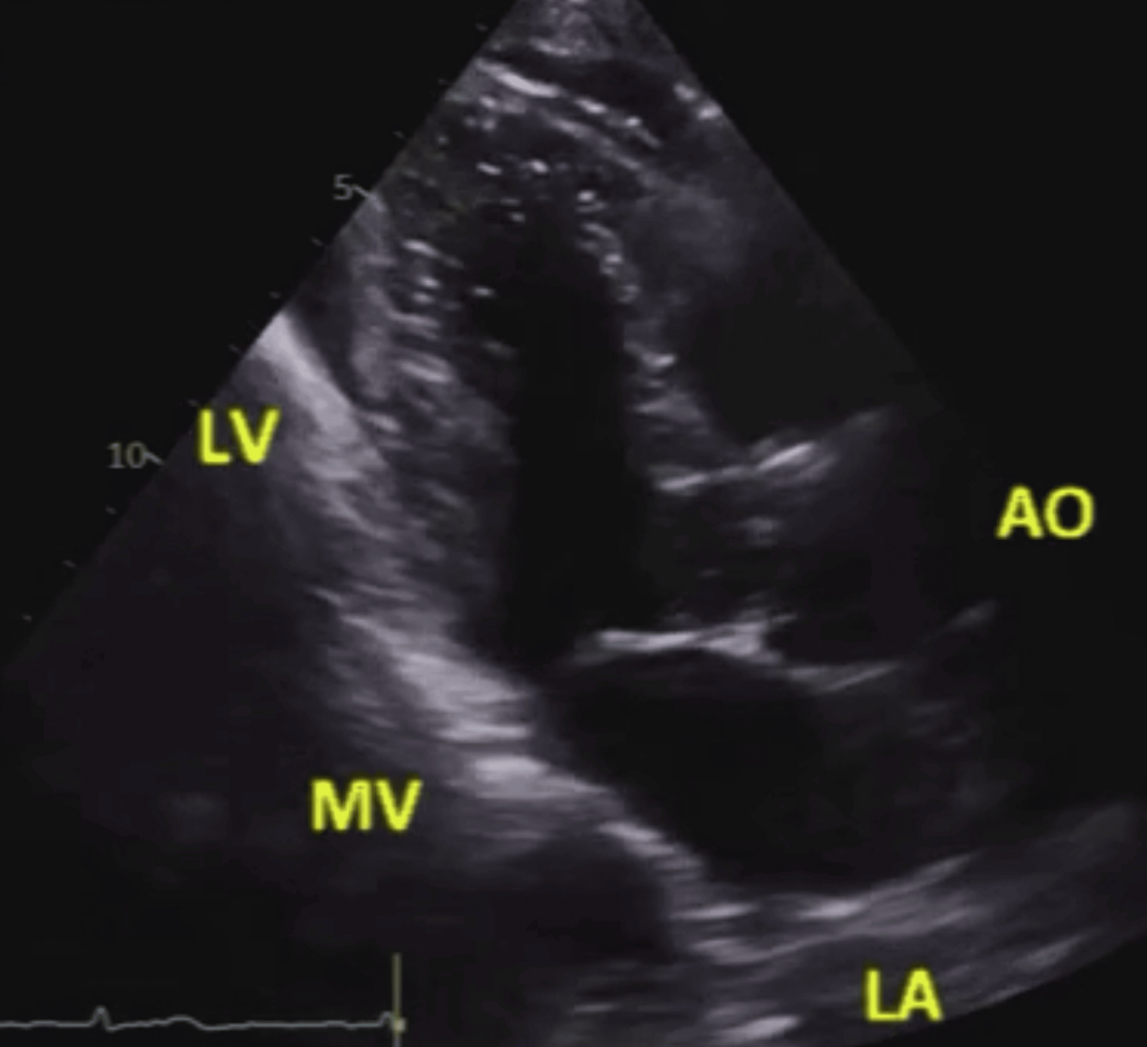
Two-dimensional echocardiography, parasternal long-axis view (off-axis), showing a static frame in systole Mitral annular disjunction and anterior mitral valve prolapse are not clearly recognized. LV: left ventricle; LA: left atrium; MV: mitral valve; AO: aorta

Other valve pathologies could not be identified throughout the scan. No obvious MAD or TAD was noted in TTE, as shown in Figures 1-2 and Videos 2-3.

**Video 2:** Two-dimensional echocardiography, apical four-chamber view, illustrating the movement of the annuli during the whole cardiac cycle No disjunction was noted.

**Video 3:** Two-dimensional echocardiography, parasternal long-axis view (off-axis), demonstrating the motion of the mitral annulus throughout the whole cardiac cycle Mitral annular disjunction or anterior mitral valve prolapse could not be identified.

**Figure 3:**
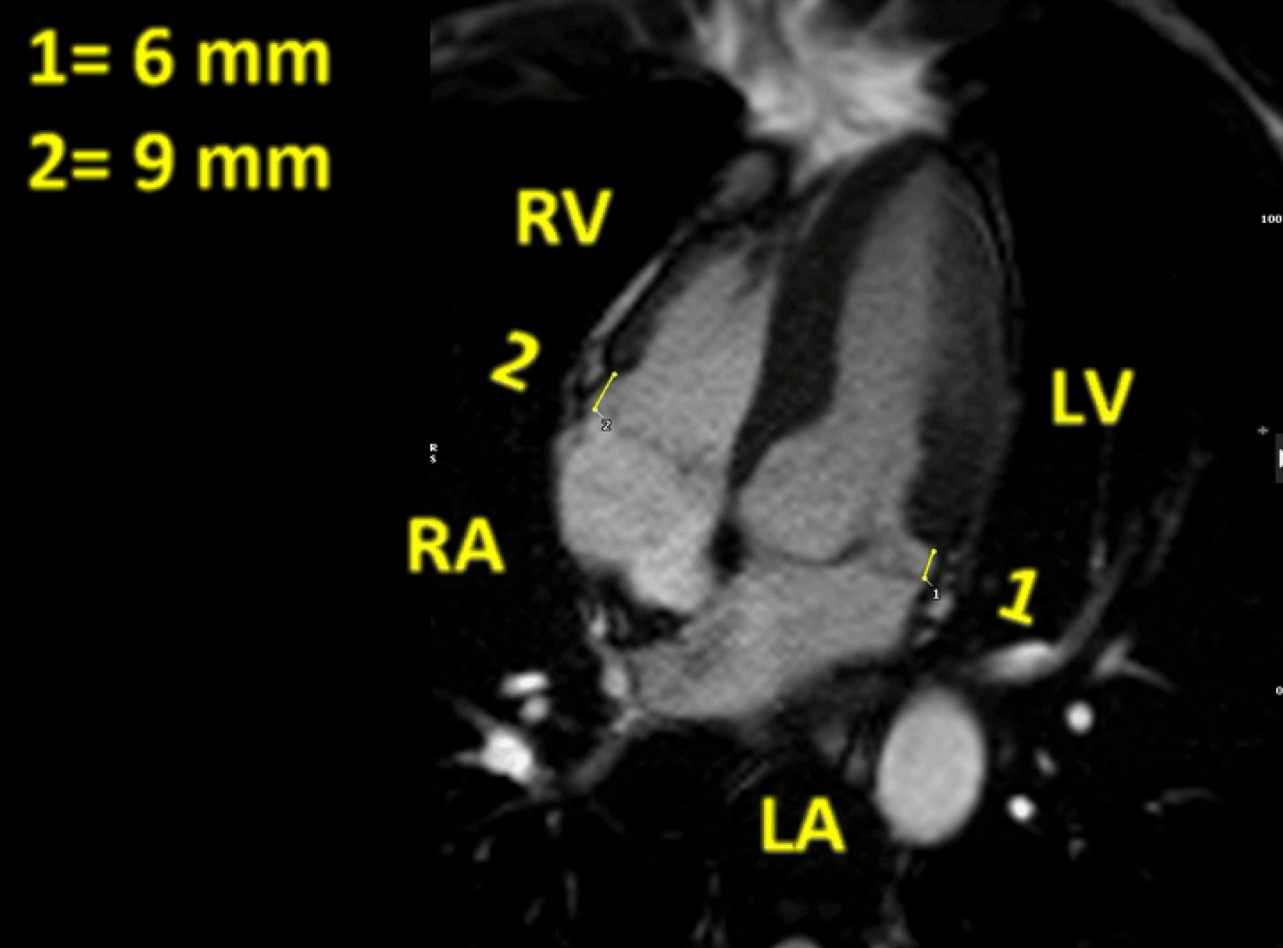
Cardiac magnetic resonance, bSSFP four-chamber cine image, showing a maximum displacement of the posterior mitral annulus during systole, of 6 mm (1), and also demonstrating a similar displacement of the tricuspid annulus with a maximum of 9 mm (2) bSSFP: balanced steady-state free precession; RV: right ventricle; LV: left ventricle; RA: right atrium; LA: left atrium

**Figure 4:**
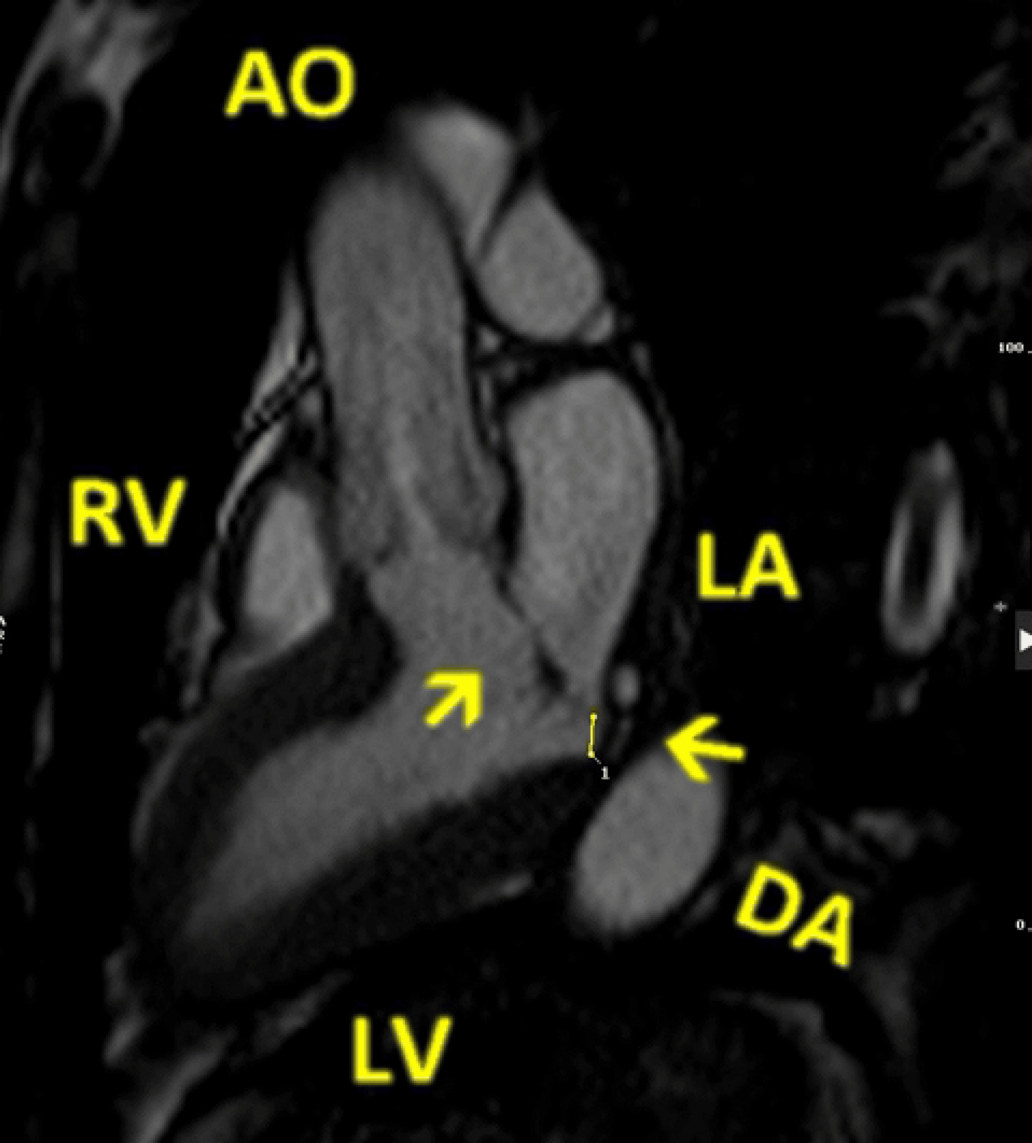
Cardiac magnetic resonance, bSSFP cine images, three-chamber view, showing a posterior mitral valve leaflet displacement (arrow pointing to the left) and a prolapse of the anterior mitral valve leaflet (arrow pointing up and right) bSSFP: balanced steady-state free precession; RV: right ventricle; LV: left ventricle; LA: left atrium; DA: descending aorta; AO: aorta

Holter ECG was recorded for seven days, which showed a sinus rhythm with episodes of sinus tachycardia: five episodes of supraventricular ventricular tachycardia (SVT), occasional episodes of trigeminy and bigeminy of polymorphic shape, as well as one episode of bradycardia. The slowest was 41 bpm, lasting four beats. Therefore, the patient was scheduled for a CMR scan to assess any underlying cardiac pathology. CMR study showed MVP of the anterior leaflet, displacement of the mitral annulus with a maximum distance of 6 mm, and TAD with a maximum displacement of 9 mm, without tricuspid valve prolapse, as shown in Figures 3-4 and Video 4.

**Video 4:** Cardiac magnetic resonance showing the motion of both annuli The disjunction is clearly noted on both sides.

**Figure 5:**
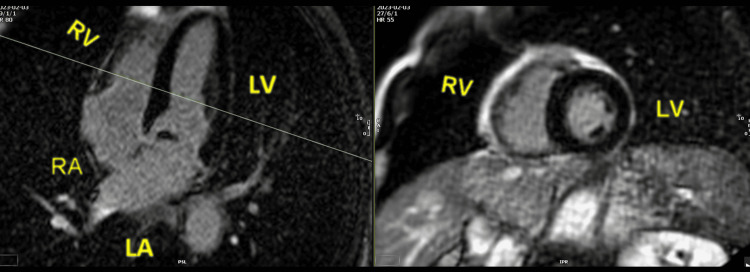
Cardiac magnetic resonance. Motion-corrected images. Late gadolinium enhancement On the left-hand panel, the cutting plane passes through the proximal portion of the papillary muscles, showing normal appearance in short axis (right-hand panel) with no evidence of fibrosis. RV: right ventricle; LV: left ventricle; RA: right atrium; LA: left atrium

**Figure 6:**
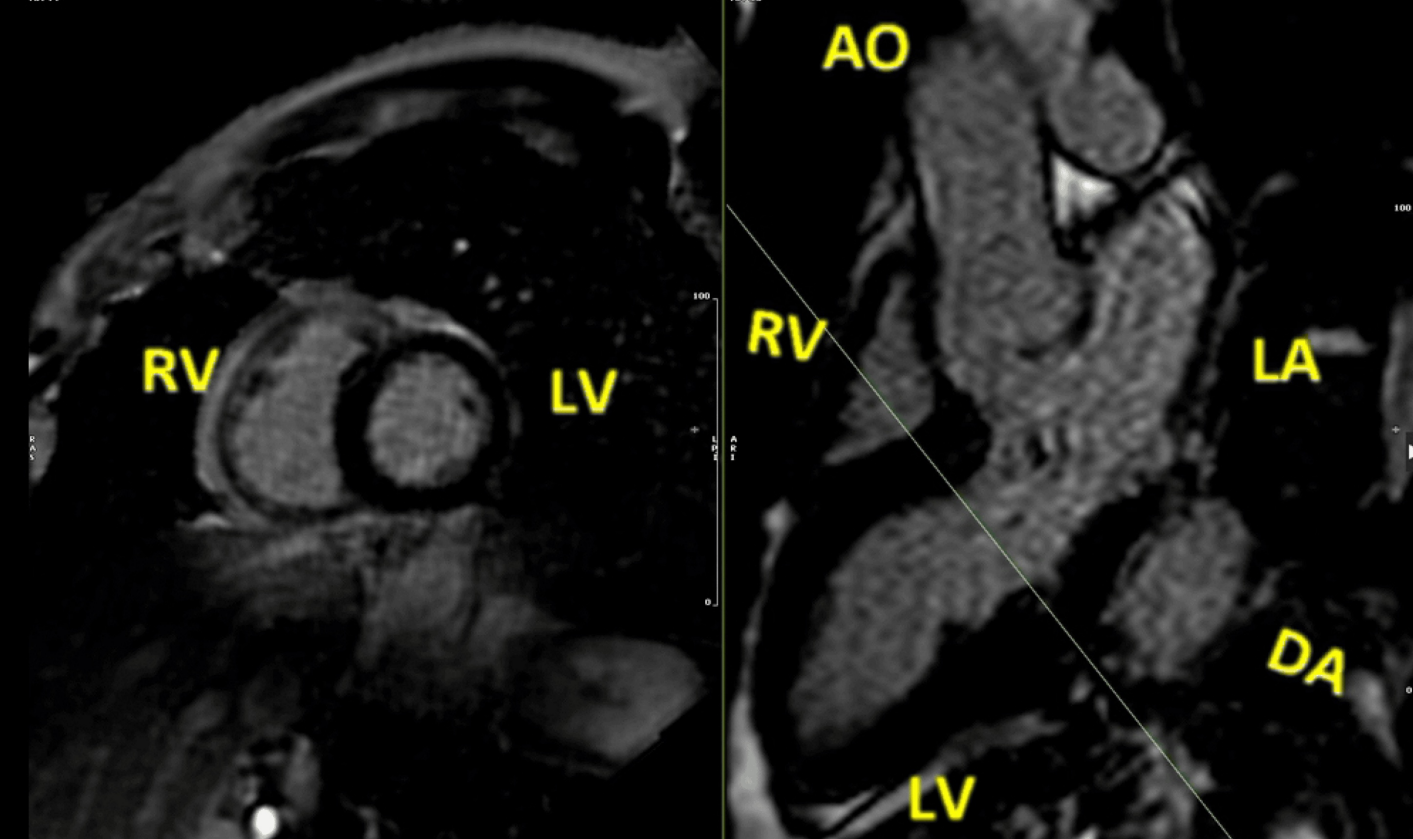
Cardiac magnetic resonance. Motion-corrected images. Late gadolinium enhancement On the left-hand panel, the plane cut through the basal inferolateral segment in apical three-chamber view, showing no enhancement in the corresponding segment in short-axis image (right-hand panel). RV: right ventricle; LV: left ventricle; LA: left atrium; AO: aorta; DA: descending aorta

Systolic curling of the LV posterior ventricular wall was also visualized. There was no evidence of myocardial fibrosis and no late gadolinium enhancement in the basal inferolateral wall or elsewhere, as seen in Figures 5-6. The etiology of the presentation was revealed, and the patient was scheduled for a rapid consultation by the electrophysiology heart team. Beta-blocker (bisoprolol) was commenced, and regular follow-up in the arrhythmia clinic was warranted. A six-week loop recorder reading demonstrated 68 episodes of narrow complex tachycardia with a maximum rate of 222 bpm, accompanied by one pre-syncopal episode.

## Discussion

The arrhythmic mitral valve complex is defined by the presence of MVP (with or without MAD), combined with frequent and/or complex VA after the exclusion of other causes [12]. Those VAs commonly arise from the outflow tract, mitral annulus, or LV myocardial papillary muscles [13,14]. The most widely recognized arrhythmogenic and phenotypic risk factors are female gender, young age, leaflet prolapse, redundant leaflet, moderate to severe mitral regurgitation, T-wave inversion, QRS prolongation in ECG, and LV myocardial or papillary muscle fibrosis [15-18]. Echocardiography could be used as an initial first-line workup to identify morphological abnormalities of the mitral valve and annulus and assess mitral regurgitation severity, which was proven non-diagnostic in our patient. The Pickelhaube sign is another marker characterized by a systolic high-velocity signal on the lateral mitral annulus by tissue Doppler and is strongly correlated with VA on Holter [19]. The latter sign has not been noted in our study though. CMR could provide a superior tool to diagnose valvopathy, quantify the severity, and identify the presence of MAD. Late gadolinium enhancement, in the basal inferolateral myocardial wall or papillary muscle, would correlate well with VA [5]. Additionally, a recent study suggests that increased extracellular volume (ECV), which is a marker for interstitial fibrosis, could be highly related to VA [20]. In general, implantation of an intracardiac defibrillator (ICD) is reserved only for cardiac arrest survivors or high-risk patients with ventricular tachycardia (VT). High-risk VT is defined as the presence of sustained VT or fast non-sustained VT >180 bpm which results in syncope. On the other hand, frequent monitoring and loop recording are recommended for patients with no high-risk VT or those with phenotypic ECG and cardiac imaging risk features [12].

## Conclusions

This case illustrates the incremental diagnostic value of CMR in evaluating AMVD phenotype when echocardiography is non-diagnostic. CMR identified bilateral annular disjunction and MVP that were not detected on initial TTE, enabling appropriate risk stratification and guiding management decisions. In patients presenting with syncope and arrhythmias where echocardiographic evaluation is limited or inconclusive, CMR may provide valuable structural and tissue characterization to inform clinical management.
